# Minimally invasive implantation and decreased inflammation reduce osteoinduction of biomaterial

**DOI:** 10.7150/thno.39507

**Published:** 2020-02-18

**Authors:** Zifan Zhao, Qin Zhao, Bin Gu, Chengcheng Yin, Kailun Shen, Hua Tang, Haibin Xia, Xiaoxin Zhang, Yanbing Zhao, Xiangliang Yang, Yufeng Zhang

**Affiliations:** 1 State Key Laboratory Breeding Base of Basic Science of Stomatology (Hubei-MOST) and Key Laboratory of Oral Biomedicine, Ministry of Education, School and Hospital of Stomatology, Wuhan University, Wuhan 430079, China.; 2 Department of Dental Implantology, School and Hospital of Stomatology, Wuhan University, Wuhan, 430079, China.; 3 Institute of immunology, Shandong First Medical University & Shandong Academy of Medical Sciences, Taian 271000, China.; 4 National Engineering Research Center for Nanomedicine, Hubei Key Laboratory of Bioinorganic Chemistry and Materia Medica, College of Life Science and Technology, Huazhong University of Science and Technology, Wuhan 430074, China.; 5 Medical Research Institute, School of Medicine, Wuhan University, Wuhan 430071, China.

**Keywords:** osteoinduction, biomaterials, T cells, MSCs, CCL5, NF-κB pathway

## Abstract

Surgical trauma of biomaterial implantation significantly influences the immune system and the biological effects of biomaterials. Minimally invasive surgery has become a trend of clinical development but violating the concept of osteoimmunomodulation will hinder the biological effects of materials. Our study focused on biphasic calcium phosphate (BCP), the ectopia osteoinductive materials, filling the research blank of the significance of adaptive immunity crosstalk with bone biomaterials, and improving the interaction mechanism between bone biomaterials and immune response.

**Methods:** The BCP bioceramics were implanted by conventional and minimally invasive methods in the gastrocnemius wild-type or T cells depleted mice to test the effect of ectopia osteoinduction. Moreover, flow cytometry was used to detect immune responses, T cell sorting and Western Blot molecular biology experiments, and transwell assays migration of mesenchymal stem cells (MSCs).

**Results:** We found that BCP, an implantable osteoinductive material, could not activate the adaptive immune response mediated by T cells after minimally invasive surgery. Further studies revealed that under the conventional non-minimally invasive BCP implantation, a positive correlation existed between T cell recruitment and the infiltration and osteogenic differentiation of MSCs. Interestingly, after BCP was implanted by minimally invasive surgery or implanted in T cell depleted mice, MSCs infiltration and osteogenic differentiation were significantly reduced, and BCP could not achieve the biological effects of ectopia ossification. Finally, we confirmed that a certain extent inflammatory stimulation activated the adaptive immune response mediated by T cells, up-regulated the nuclear factor-κB (NF-κB) signal in T cells, released a large amount of chemokine C-C motif chemokine ligand 5(CCL5) to recruit MSCs to the surrounding material, and finally achieved the ideal effect of osteoinduction.

**Conclusion:** From experimental research and clinical surgery, this study discovered that the T cells are indispensable in the ectopia ossification mediated by osteoinductive materials, put forward and confirmed the surgery method as a key variable factor restricting the application effect of biological materials, enriched the key mechanism of adaptive immunity in osteoimmunomodulation, and laid a theoretical foundation for the development of osteoinductive materials and bone tissue regeneration.

## Introduction

The application of implantable biomaterials is the primary means of new bone formation and bone regeneration in the field of bone engineering^1^, ^2^. With the progress of biomaterial medicine, the research and development of new osteoinductive materials play an important role in solving the problems of bone regeneration^3^, emphasizing on the transformation from experimental research to clinical application^4^. Regardless of the material implantation of experimental models *in vivo* or biomaterial implantation in clinical bone regeneration, the surgical trauma of local tissues inevitably occurs. Experimental and clinical studies have found that surgical trauma significantly affects the immune system, including the innate and adaptive immune responses^5^. In addition, the implantation of biological materials is accompanied by a series of osteoinduction immune response events. After the implantation, the immune system recognizes biological materials as a foreign body which triggers the acute inflammatory response mediated by innate immune cells; through inflammatory hyperemia and cellular exudation, phagocytes devour and eliminate the danger signals while recruiting larger quantities of inflammatory cells to induce innate and adaptive immune responses^6^-^8^. Therefore, the immune response induced by surgery and implants jointly determines the ultimate fate of biological materials in the complex environment of the body.

Osteoimmunomodulation provides a valuable strategy for the development of advanced bone biomaterials^9^. However, the interference of surgical methods on the immune response is often neglected. Uniform and standardized surgical procedures are important prerequisites for standardized laboratory and clinical studies to obtain reliable scientific conclusions and implement rational treatment regimens. Minimally invasive surgeries that cause less trauma, such as heart valve surgery, plastic surgery, and periodontal treatment, have been widely used in clinical practice to reduce the local or systemic immune response and wound surface^10^-^12^. However, inflammatory responses are not all negative responses, and appropriate inflammation promotes the biological effects of biomaterials^13^. Neutrophils and mononuclear macrophages around the biomaterial implantation bed secrete cytokines and growth factors, such as vascular endothelial growth factor-α(VEGF-α), to promote angiogenesis, stimulate the proliferation of fibroblasts, epithelial cells, and other cells, and participate in guiding vascular remodeling^14^. Therefore, from the perspective of immunology, minimally invasive surgery may be unfavorable to the performance of biomaterials in several cases.

With tissue damage and material implantation, the first phase of the immune response is initiated, and it involves components of the innate immune system that provide immediate defense against potential pathogens invading damaged tissues. However, the initial immune response triggered by inflammatory signals from the damaged tissue can cause aseptic inflammation despite the absence of pathogens^15^, ^16^. Most researchers suggest that innate immune response is an important immune stage regulating tissue healing and regeneration^17^. A mild inflammation will terminate the immune response process, whereas an extensive inflammation will activate the adaptive immune system, which is often undesirable in tissue regeneration^18^. However, T cells, as the key cells in adaptive immunity, are recruited and activate the STAT3 signaling pathway after stimulation by inflammatory signals, leading to the increased release of chemokine CCL5 and recruitment of MSCs to the site of injury^19^, ^20^. The recruited MSCs around materials are important for the regeneration function of biomaterials^21^. Although the superficial (primary) adaptive immune response is considered a secondary immune stage or an adverse factor in tissue healing, T cell activation and multifunctional subsets may play key roles in tissue repair and regeneration.

Most current research focus on how transplanted MSCs regulate T cell activity and immune tolerance 22, 23. However, the understanding of how T cells regulate tissue resident stem cells and tissue healing process is still in the primary stages. Here, the surgical method we established is intrinsically related to the T cell immune response, and it has been proven that minimally invasive surgery and T cell depletion will lead to insufficient local recruitment of MSCs. Eventually, the bone biomaterials cannot complete the osteoinduction function. In this study, we revealed the important role of T cells in the process of biomaterial-mediated tissue regeneration, proposed and confirmed the influence of surgical methods on biomaterial experiments and clinical studies, and provided a theoretical basis for the rational utilization of surgical trauma to optimize tissue regeneration.

## Materials & Methods

### Ethical Approval

The mice utilized in this study were treated according to the policy of the Ethics Committee for Animal Research, Wuhan University, China. Proper sterile conditions and minimally invasive surgical procedures were utilized throughout the entire surgery and the study protocol was approved by the Ethics Committee for Animal Use of the Institute of Biomedical Sciences (Protocol number 69/2017).

### Preparation of biphasic calcium phosphate (BCP)

The BCP we used were made with a HA/β-TCP ratio of 60/40, irregular particles of 0.6mm in diameter and all BCP have a uniform porosity and specific surface area. The particles of BCP were synthesized by wet chemical precipitation. First, 0.24 mol ammonium dihydrogen phosphate ((NH_4_)_2_HPO_4_, AR) solution was dropped into 300 ml calcium nitrate (0.36 mol; Ca(NO_3_)_2_·4H_2_O, AR) solution and stirred vigorously. The temperature was raised to 55 ℃. After agitation, ammonia (NH_3_·H_2_O, 25%-28%, AR) was added to adjust the pH of the system to 7.5, and the system was stirred for another 2 h. The white suspension was aged for 36 h at room temperature after precipitation. The white product was centrifuged, washed with deionized water, and dried in air at 80°C for 12 h, whereas the powder was calcined at 1100°C for 3 h. Scanning electron microscopy (SEM) micrographs **(A, B)** and X-ray diffraction (XRD) **(C)** for BCP granules were demonstrated in **(Supplementary Fig. 1)**. Macroscopic photo show dimensions of BCP granules **(Supplementary Fig. 1D)**, BCP granules approximately 0.6mm in size were selected during implantation.

### Mouse experimental model

**Figure 1:**
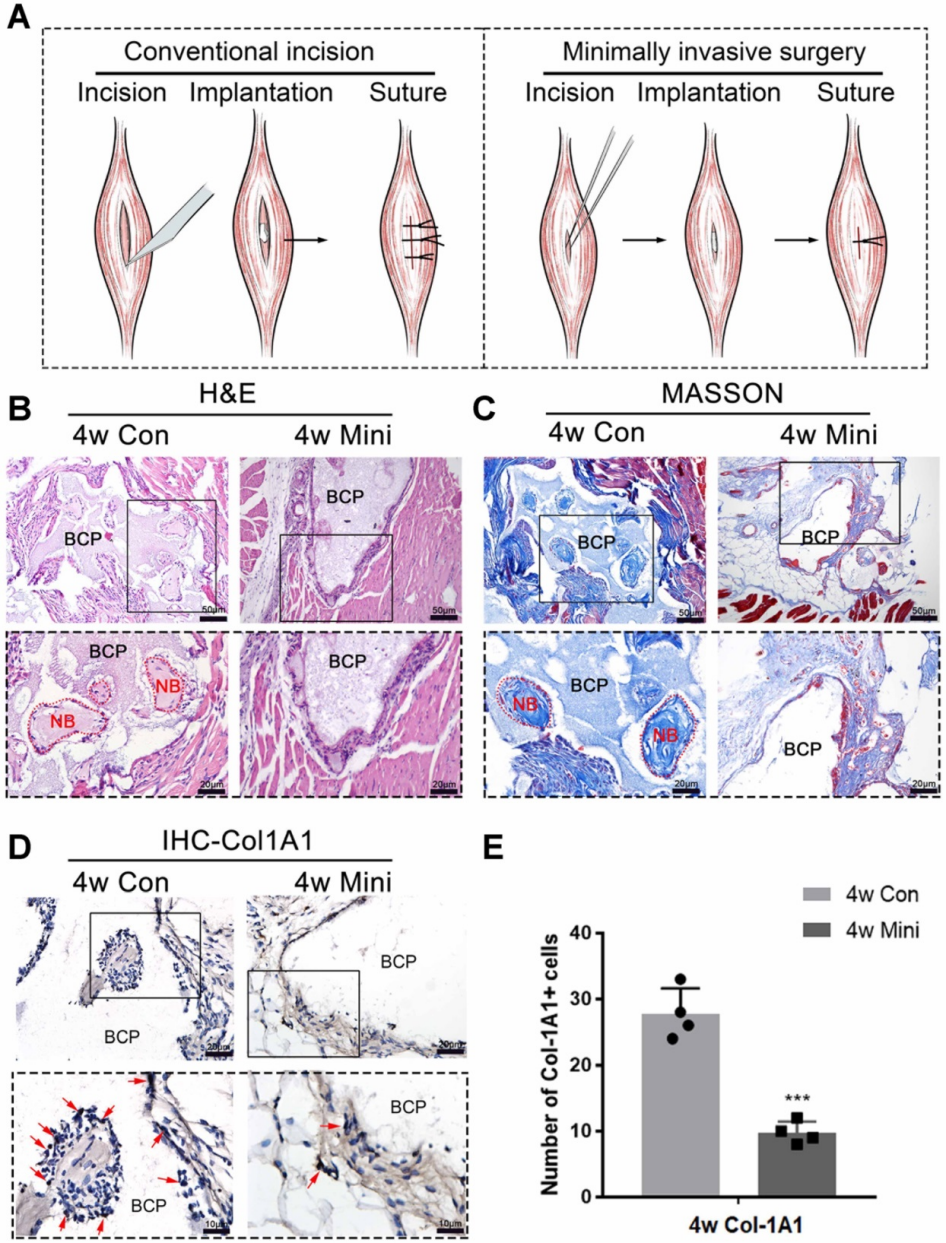
Surgical trauma affected the osteoinductivity of BCP. (**A**) The methods of appropriate traumatic conventional incision and almost nontraumatic minimally invasive surgery. (**B-D**) BCP was implanted into mice by the conventional incision and minimally invasive surgery respectively for 4 weeks. (**B, C**) H&E and Masson staining showed the formation of new bone. New bone (NB) area is circled by the red dotted line. (**D**) IHC of Col-1A1 showed the expression of osteogenic indicator marked by red arrows. (**E**) Quantitative comparison of positive Col-1A1 expression. (n=4) ***P < 0.001.

Female C57BL/6 mice aged 8 weeks were selected for the animal experiment. The conventional implantation method involves making an incision on the skin and muscle with a scalpel after the preoperative hair removal. The incision approximated 8 mm in length and was made with precision to avoid damaging the blood vessels. The BCP particles were inserted into the wound using ophthalmic forceps and sutured to the muscle layer and skin. The minimally invasive implantation method involves making a skin incision with a length of about 2 mm using a sharp knife after preoperative hair removal. Microtweezers were used to separate the muscle, and a muscle gap with 2 mm width was created. The BCP particles were inserted into the gap, and the skin was sutured with a needle. The operation process was shown in **(Supplementary Fig. 2)** and the schematic diagram was shown in **(Fig. 1A)**.

### Histological staining

After fixing with 4% formaldehyde for 24 h, the muscle samples were immersed in decalcified liquid, which was changed frequently, added with 10% EDTA (Ethylene Diamine Tetraacetic Acid), subjected to 4 weeks of decalcification, and then gradient-dehydrated for embedding in paraffin. Hematoxylin and eosin (H&E), Masson, and immunohistochemistry staining were performed following the manufacturer's protocols to detect ectopia ossification (MXB Biotechnologies, China). The immunohistochemistry staining primary antibodies included the following: Runx2 (1:800; Abcam ab76956, Cambridge, UK), CD146 (1:150; ABclonal A9985, China), and Col-1A1 (1:400, ABclonal A2357, China). For immunofluorescent staining, the sections were incubated with primary antibodies of CD4 and the secondary antibody with green fluorescent marker (Abbkine, U.S.A). The cell nuclei in tissues were stained using 4ʹ,6-diamidino-2-phenylindole dye(DAPI) (Zhongshan Biotechnology, Ltd, China) in the mounting medium. The images of all stained sections were captured with C. Yin et al./Acta Biomaterialia 73 (2018) 522-530 523 an Olympus DP72 microscope. For the quantification of immunohistochemically positive expression cells in tissues, we randomly selected five equal areas around the materials in one figure, calculated the number of these cells in the selected area, and finally performed statistical analysis.

### Flow cytometry of mouse muscle tissues

After digestion with collagenase II (2 µg/mL) for 45 min at 37 °C, the same-weight muscle samples were filtered by a 220 µm filter, washed with phosphate-buffered saline (PBS), centrifuged at 2500 rpm for 5 min, and then transferred to a 96-well plate. The antibodies used are as follows, CD45-PE, CD3-A780, NK1.1-APC, TNFα-PE, IL6-APC (Biolegend, U.S.A).

Flow cytometry was performed using a BD Biosciences LSR Fortessa flow cytometer, and the data were analyzed using FlowJo 10.

### CD4+ and CD8+ T cells deletion

Hybridoma cell line GK1.5 and 2.43 were cultured in Roswell Park Memorial Institute (RPMI)-1640 (Hyclone) supplemented with 10% fetal bovine serum (FBS; Gibco, Life Technologies Corporation) at 37 °C under humid conditions with 5% CO_2_. After the cell density reached 80%, the cells were cultured for additional 24 h, and the supernatant was collected after centrifugation. Deletion efficiency was determined by immunofluorescence **(A, B)** and flow cytometry **(C, D)** and the results were presented in** (Supplementary Fig. 5)**, up to the experimental standard.

### Cell culture

Mice bone marrow stem cells were cultured in a-Minimal Essential Medium (MEM) (Hyclone) supplemented with 10% FBS and 100 U/mL penicillin G and 100 mg/mL streptomycin (Hyclone) at 37 °C under humid conditions with 5% CO_2_. In addition, the NF-κB pathway was blocked by pyrrolidine dithiocarbamate (PDTC) (Selleck, Houston, TX, U.S.A).

### T cells sorting

The spleen of 8w healthy female C57BL/6 mice was selected to prepare single cells and erythrocyte lysis. T cells were sorted by EasySep Mouse Biotin Positive Selection Kit II (Stemcell, 17663, U.S.A). In brief, the CD3+ T cells were marked by CD3-Biotin antibody (BioLegend, U.S.A) and connected with beads, adsorbed to the wall of the test tube in the magnetic field and washed down after discarding the supernatant, maintained in lymphocyte culture medium (Hyclone, U.S.A) containing 10% FBS and other culture conditions as previously described.

### Protein extraction and Western blot

RIPA lysate containing phenylmethylsulfonyl fluoride (PMSF) (1 mM) was added to the cells, and the resulting sample was then vibrated in an ultrasonicator for complete cell lysis. After mixing with the loading buffer, the protein samples were heated at 95 °C for 10 min for denaturation, separated by sodium dodecyl sulfate polyacrylamide gel electrophoresis(SDS-PAGE), and transferred to nitrocellulose membranes (0.45 µm). The membranes were soaked in 5% non-fat milk for 1 h and incubated overnight with primary antibodies against CCL5 (1:1000, ABclonal, Wuhan, China), NF-κB p65 (1:1000, CST, U.S.A.), phospho-NF-κB p65 (1:1000, CST, U.S.A.), or GAPDH (1:5000, Proteintech, U.S.A.) at 4°C. In the following day, secondary antibodies (BioSharp, Hefei, China) were incubated for 1 h and visualized by WesternBright ECL HRP Substrate Kit (Advansta, U.S.A.).

### Transwell migration assays

Mouse BMSCs were starved cultured with 2% FBS α-MEM medium for 12 h and inoculated in 24-well transwell chambers (5×10^3^ cells per chamber). Six transwell chambers were assigned to a group and cultured with the four groups of T cells in a 24-well plate for 24 h. The cells were fixed in 4% formaldehyde for 15 min, stained with crystal violet (Beyotime, Biotechnology, China), and washed with PBS three times. Images were captured.

### Statistical analysis

GraphPad Prism software 6.0 (GraphPad, San Diego, CA, USA) was used for data analysis. The differences between the two groups were evaluated through two-way ANOVA and students' t-test. P-values less than 0.05 were considered as statistical significance.

## Results

### Minimally invasive surgery interferes with ectopia ossification of BCP

Conventional and minimally invasive methods were used to implant BCP in the gastrocnemius muscles of mice to demonstrate the inhibition of minor surgical trauma during implantation on the osteoinductive effects of bone biomaterials. The effect of ectopia osteogenesis was observed after 4 weeks. In the conventional incision group, a scalpel was used to cut through the gastrocnemius to create a larger wound to implant BCP (15mg/sample). In the minimally invasive group, only the skin was incised, muscle fibers were separated by blunt microtweezers, and equivalent BCP was embedded in the gap **(Fig. 1A)**. Histological staining was performed 4 weeks after the operation. HE and Masson staining showed that new bone was formed in the conventional incision group, however, no new bone formation was found in all samples of the minimally invasive surgery group **(Fig. 1B-C) (n=4)**. Immunohistochemistry was used to detect Col-1A1, an osteogenic indicator. The positive expression of Col-1A1 in the minimally invasive surgery group was significantly lower than that in the traditional surgery group **(Fig. 1D-E)**. New bone formation occurred at 4 weeks in the conventional incision group but not in the minimally invasive group.

### Inflammation level in the conventional incision group is higher than that in the minimally invasive group

Multiple time points from post-implantation to pre-osteogenesis were selected to further explore factors that lead to completely different results for the same material implanted by different surgical methods. The major difference between minimally invasive surgery and conventional surgery is the level of trauma, so we examined the extent of the local immune response to explore the reasons why the former interferes with the ectopia ossification of BCP.

**Figure 2:**
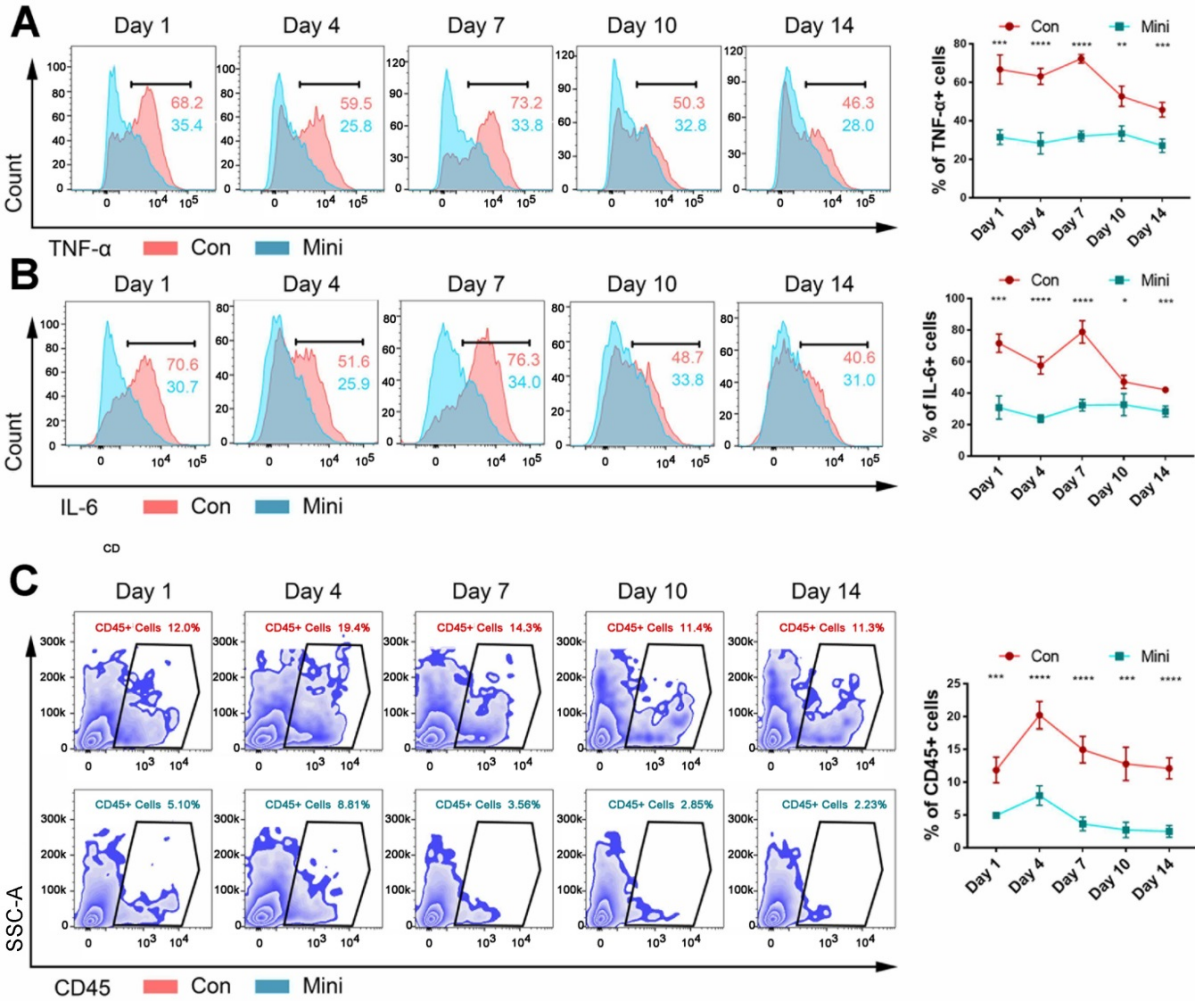
The difference between the two methods of surgery was the level of inflammation. (**A, B**) The levels of TNF-α (**A**) and IL-6 (**B**) were detected by flow cytometry after BCP implantation 1,4,7,10,14 days. (**C**) The counts immune cells were detected by flow cytometry after BCP implantation 1,4,7,10,14 days. (n=4) ****P < 0.0001. ***P < 0.001. ** P < 0.01. * P < 0.05.

After 1, 4, 7, 10, and 14 days, flow cytometry was used to determine the number of inflammatory cytokines and immune cells in the tissues of the two groups. Within 1-14 days, the inflammation level **(Fig. 2A-B)** and the number of CD45+ immune cells **(Fig. 2C)** in the conventional incision group were significantly higher than those in the minimally invasive group. Hence, immune response plays an important role in the development of biological effects of bone-induced materials. The failure of these materials could be due to too low or lack of immune response. Additionally, CD45+ immune cells in normal muscle tissue were showed in **Supplementary Fig. 3**.

### Inflammatory T cells are correlated with mesenchymal cell infiltration

**Figure 3:**
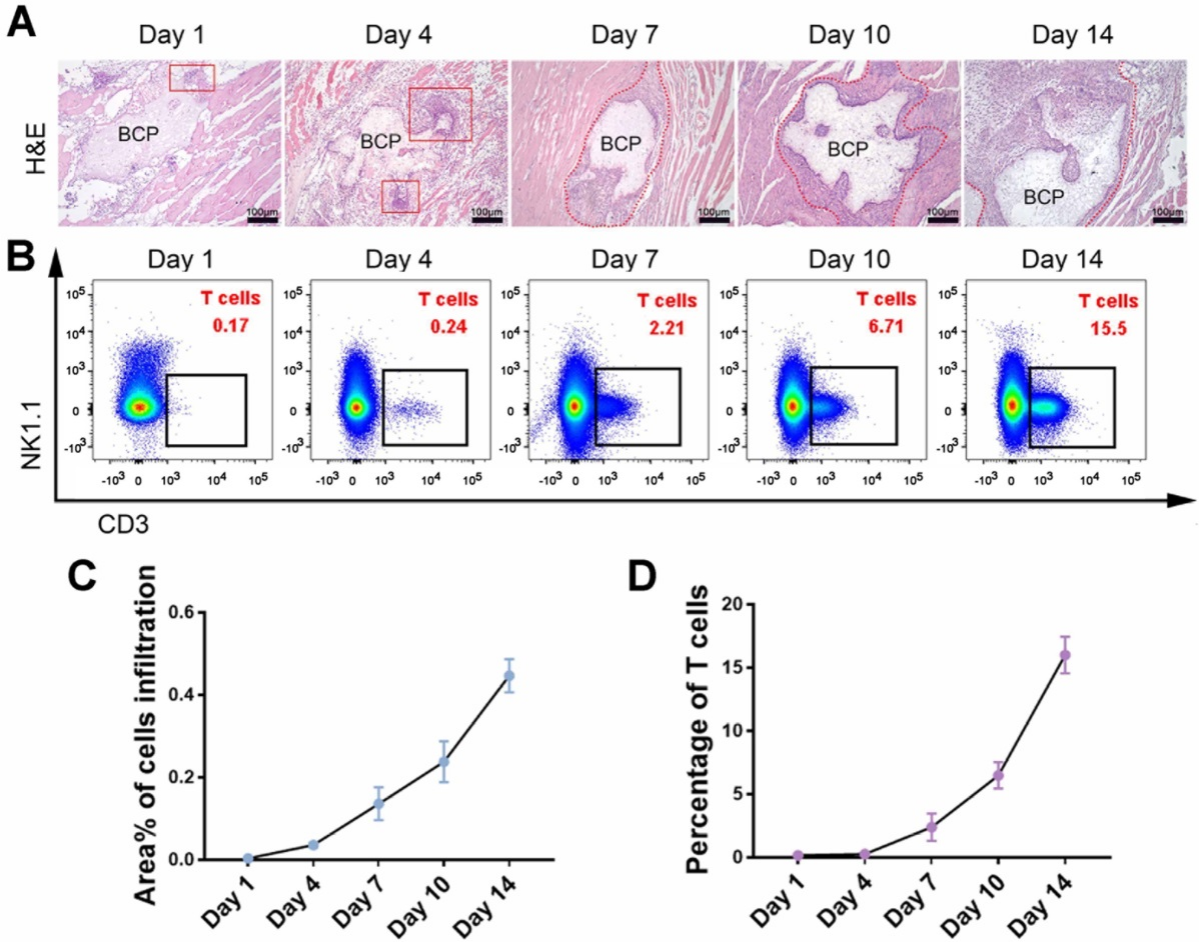
T cell was the type of immune cell related to the total number of infiltrating cells around BCP. (**A**) H&E staining showed the amounts of infiltrating cells around BCP. Infiltrating cells were circled by the red line. (**B**) The number of T cells around BCP was detected by flow cytometry. (**C, D**) Infiltrating cells (**C**) and T cells (**D**) were quantified respectively, infiltrating cells grow rapidly in the presence of T cells. (n=4)

Infiltration of mesenchymal cells, including mesenchymal stem cells (MSCs), is critical in ectopia osteogenesis. We conducted further studies on conventional incision group to explore responses before osteogenesis. The infiltration of mesenchymal cells was observed around BCP at 1-14 days, and the number of cells increased significantly at 7-14 days **(Fig. 3A, C)**. The larger magnification images to observe the infiltrating cells in **Supplementary Fig. 4**. The presence of various immune cells around BCP was detected by flow cytometry to investigate which immune cells interfere with ectopia osteogenesis. Monocytes, neutrophils, and macrophages, which represent innate immunity, were mainly present at 1-3 days. T cells, which represent adaptive immunity, were mainly present at 7-14 days **(Fig. 3B, D)**. These results suggest that the infiltration of mesenchymal cells around BCP is probably related to T cells.

### Recruited cells associated with T cells have the ability to ossify

**Figure 4:**
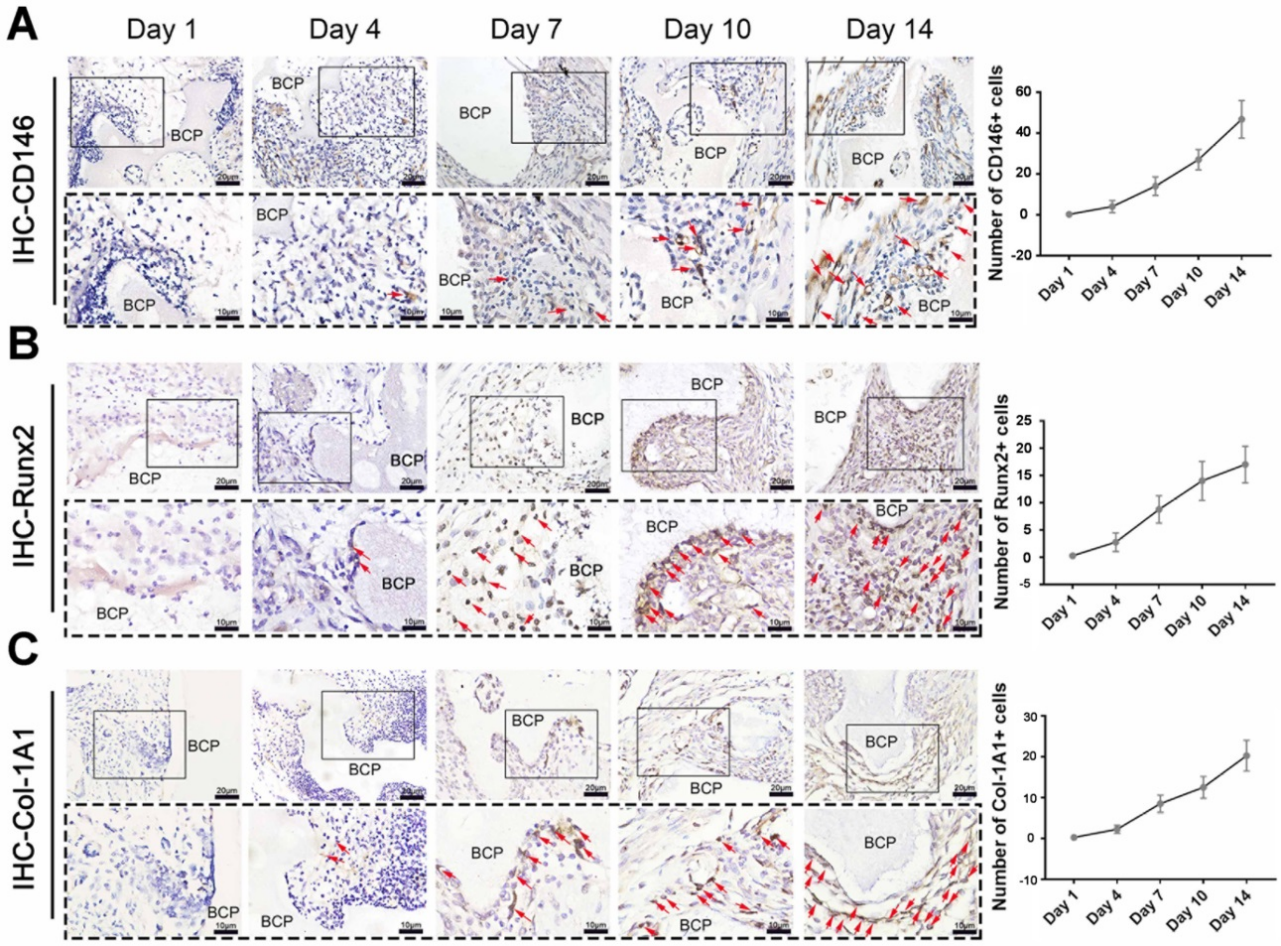
Osteogenic differentiation of MSCs around BCP increased at 7-14 days.CD146 (**A**), Runx2 (**B**), Col-1A1 (**C**) were detected and quantified by IHC staining. (n=4)

MSCs, which are capable of osteogenic differentiation, are the most relevant cells to ectopia ossification. The content of key cells, namely, MSCs that underwent osteogenic differentiation, was detected in the mesenchymal cells around the materials. A variety of osteogenic indicators were used for immunohistochemical staining. The number of cells with positive expression of CD146 **(Fig. 4A)**, Runx2 **(Fig 4. B)**, and Col-1A1 **(Fig. 4C)** significantly increased at 7-14 days. The increase is closely correlated with the time of T cell existence. Therefore, we hypothesized that T cells play an important role in the infiltration of MSCs around the materials.

### Usage and effectiveness of T cell depletion antibodies

**Figure 5:**
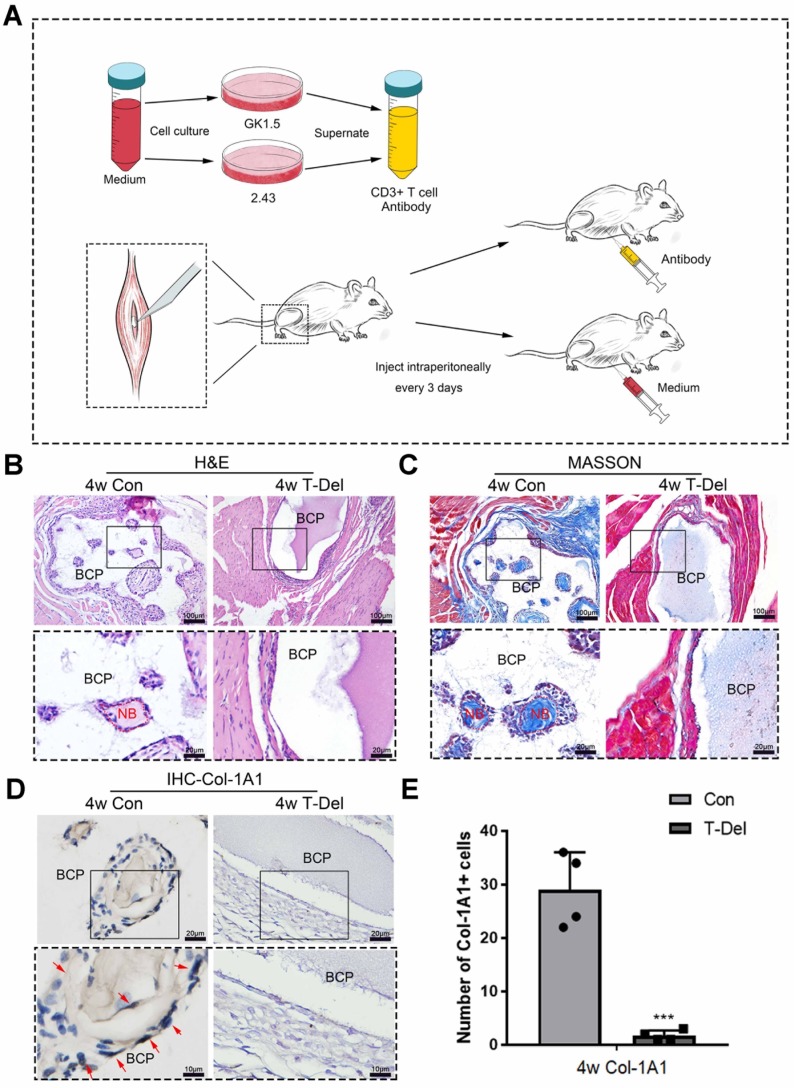
The osteoinductivity of BCP is dependent on T cells. (**A**)T cells in mice were deleted by the supernatant of hybridoma cells. (**B-D**) BCP was implanted into mice injected with T cells antibody (T-Del group) or complete medium (Con group) every 3 days for 4 weeks. (**B, C**) H&E and Masson staining showed the formation of new bone. New bone (NB) area is circled by the red dotted line. (**D**) IHC of Col-1A1 showed the expression of osteogenic indicator marked by red arrows. (**E**) Quantitative comparison of positive Col-1A1 expression. (n=4) ***P < 0.001.

To confirm our hypothesis, we used T cell deleting antibodies to clear the T cells in mice and implanted BCP into the gastrocnemius muscles. We used the culture supernatant of hybridoma cells GK1.5 and 2.43 to prepare antibodies for clearing CD4+ and CD8+ T cells. The T cell deletion group was injected with T cell deletion antibody, and the control group was injected with complete RPMI-1640 medium every 3 days after the conventional surgery **(Fig. 5A)**. CD3+T cells around BCP were detected by immunofluorescence at 7 and 14 days after the conventional surgery. After the injection of the antibody, the clearance efficiency reached more than 90% at 7 days, and almost no T cells were detected at 14 days **(Supplementary Fig. 5A, B)**. The results of FACS also confirmed that the deletion efficiency reached the experimental standard **(Supplementary Fig. 5 C)**.

### BCP loses its biological effects on the environment of inflammation without T cells

**Figure 6:**
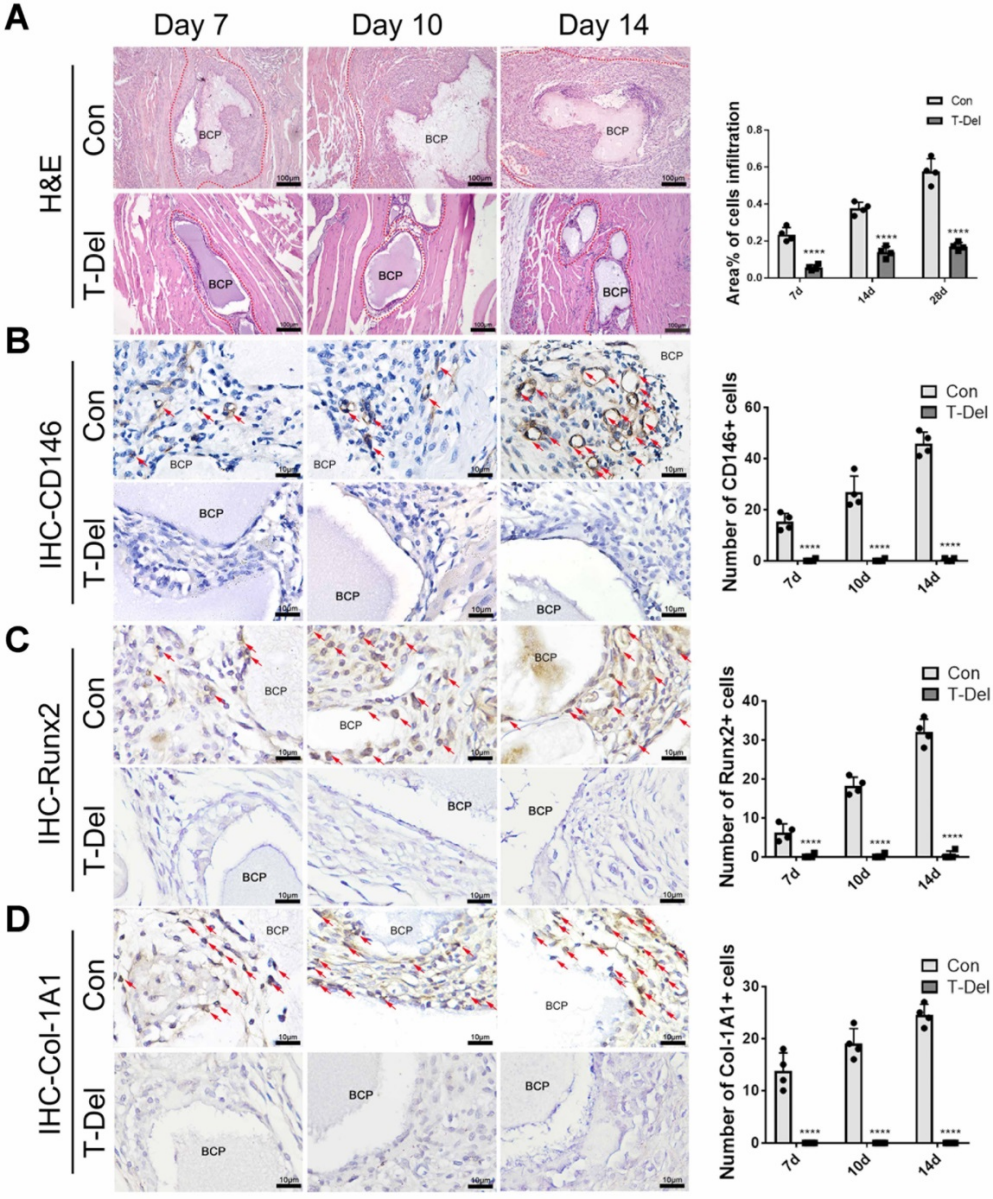
Infiltration of Cells and osteogenic differentiation MSCs around BCP dependent on T cells. (**A**) H&E staining showed cells infiltration was significantly reduced in the T-Del group. (**B-D**) IHC staining of CD146 (**B**), Runx2 (**C**), Col-1A1 (**D**) were detected and quantified by IHC staining. Positive expressions were significantly reduced in the T-Del group. (n=4) ****P < 0.0001.

To verify whether T cells are indispensable in MSC infiltration and ectopia osteogenesis, we implanted BCP into the gastrocnemius muscle. Mice in the T-Del group were injected with T-cell antibody, and those in the Con group were injected with complete culture medium (2 mL per mouse) every 3 days. Samples were collected for histological staining 4 weeks later. The results of H&E and Masson histologic staining showed that similar to that in normal mice that underwent the conventional surgery, new bones were formed in the control group injected with complete culture medium. No new bones were formed after 4 weeks of the conventional surgery without T cells **(Fig 5. B-C)**. The results of IHC showed the lower Col-1A1 expression level in the T-Del group than in the control group **(Fig. 5D-E)**. Hence, T cells are essential for the ectopia ossification of biomaterials. Further histological examination showed that the rate of mesenchymal cell infiltration at 1-14 days after the conventional surgery around BCP significantly decreased **(Fig. 6A)**. Almost no osteogenic MSCs existed in the T-Del group **(Fig. 6B-D)**. The presence of T cells within the environment of inflammation exhibited a recruitment effect on MSCs, and such effect is crucial in osteogenesis. MSCs without T cells cannot be recruited even in the environment of inflammation, and ectopia osteogenesis is ultimately impossible.

### T cell-mediated adaptive immune response recruits MSCs

**Figure 7:**
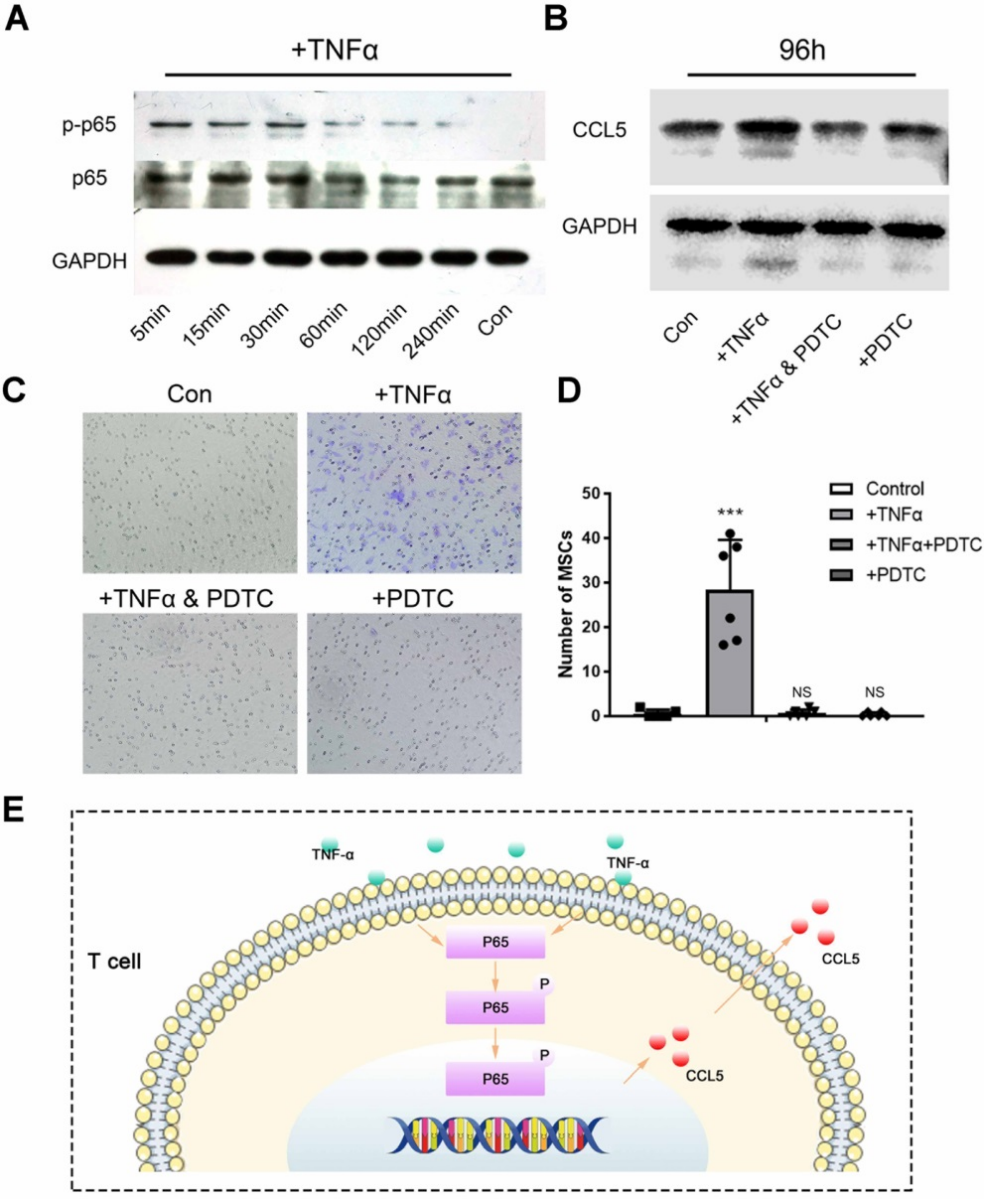
T cell-mediated adaptive immune response recruits MSCs. (**A**) Activation of NF-κB pathway in T cells after TNF-α stimulation was detected by Western Blot. (**B**) Western Blot showed the secretion of CCL5 by T cells after the NF-κB pathway was activated by TNF-α or blocked by PDTC. (**C**) Transwell image showed the chemotaxis of T cells to MSCs after the NF-κB pathway was activated by TNF-α or blocked by PDTC. (**D**) Quantitative comparison of the chemotaxis of T cells to MSCs in each group. (n=6) ***P < 0.001, NS, no statistical significance. (**E**) TNF-α activates the NF-κB pathway to increase the release of CCL5 in T cells.

To further confirm the recruitment mechanism of T cells to MSCs, we used magnetic beads to sort the spleen T cells of mice and culture them with BCP *in vitro*. TNF-α was used as an inflammatory factor to create an inflammatory environment and activate T cell inflammatory response. NF-κB, a key pathway of inflammatory response, is related to the secretion of chemokines of T cells. The Western blot showed that the NF-κB pathway in T cells was activated within 5 min to 4 h after TNF-α stimulation **(Fig. 7A)**. After the stimulation of TNF-α, the expression of chemokine CCL5 secreted was increased by T cells with BCP. Then, after addition of PDTC (the inhibitor of NF-κB pathway), the CCL5 secreted returned to the level of control group **(Fig. 7B)**. Hence, the NF-κB pathway is indispensable in regulating the CCL5 secretion of T cells. To further test the practical effect of the results, we verified the chemotaxis of T cells on MSCs by Transwell co-culture experiment. The chemotaxis of T cells on MSCs increased significantly after the addition of TNF-α and recovered to the level of the control group after blocking the NF-κB pathway **(Fig 7. C-D)**. Hence, the NF-κB pathway was activated after T cells received inflammatory stimulation, and the secretion level of CCL5 increased, thereby promoting the chemotaxis of MSCs **(Fig 7. E)**. Finally, we concluded that T cells around the materials were stimulated by inflammation. Such cells activate the NF-κB pathway, release CCL5 to recruit MSCs, and achieve the biological effect of ectopia ossification.

## Discussion

After biological materials are implanted in the body, tissue trauma caused by surgery will affect local or systemic immune response^24^. The osteoimmunomodulation of materials is considered a key feature of bone biomaterials mediating bone formation, which depends on the immune response of the body and the characteristics of bone biomaterials^25^. In research and clinical application of bone induction materials, properties, such as surface properties, particle size, porosity, and release of bioactive ions, are the focus of attention^26^-^30^. In addition, the response of the body to bone biomaterials is important and considerably determines the effect of osteoinduction^13^. In the present study, we demonstrated that appropriate surgical trauma or inflammation is beneficial to the ectopia osteoinduction of biomaterials. This pivotal osteoinduction cannot occur without the participation of adaptive immune T cells, which can accumulate around the material implantation area and release chemokine CCL5 to recruit MSCs, thereby inducing MSCs to differentiate into osteoblasts by bone biomaterials.

In mouse implant model, BCP bioceramics with osteoinduction properties were implanted in the gastrocnemius through minimally invasive surgery and conventional surgery. BCP bioceramics is a type of calcium phosphate (CaP) ceramics, including hydroxyapatite (HA) and alpha-and beta-tricalcium phosphate (alpha-TCP and beta-TCP). These materials are used as graft materials because of their good biocompatibility, safety, availability, low complication, and cost effectiveness. BCP consists of HA and β-TCP in different proportions, with the HA/β-TCP ratios of 50/50 and 60/40 lead to a significantly greater percentage of new bone formation^31^. BCP can achieve ectopia osteogenesis in muscles with conventional incision implantation, consistent with previous reports^31^, ^32^. However, BCP showed the failure of osteoinduction with minimally invasive surgery possibly due to the reduced local inflammatory levels during the minimally invasive surgery. We detected significantly lower expression of CD45+ immune cells and pro-inflammatory cytokines, namely, IL-6 and IFN-γ, in the tissues surrounding the material during the minimally invasive surgery. Previous studies showed that mesenchymal cells, such as MSCs, endothelial cells, and fibroblasts, exhibit reduced chemotaxis and proliferation rate under low inflammatory conditions, similar to the present results^33^. These mesenchymal cells, especially MSCs, are important potential cells for biomaterials to achieve osteoinduction^34^. Appropriate inflammation can ensure the occurrence of the following series of interactions between bone biomaterials and adequate MSCs^35^. First, MSCs should be recruited around the materials. Second, materials should convert undifferentiated MSCs into mature osteoblasts. Finally, materials should induce endogenous ectopia bone formation in tissues other than bone. In this process, macrophages play a crucial role in the recruitment and differentiation of MSCs. We speculated that the low level of immune response induced by minimally invasive surgical implantation inhibited the recruitment of MSCs. Hence, the osteogenic differentiation of biomaterials and sufficient MSCs could not be achieved, leading to the failure of bone induction. Local tissue and bone marrow are the source of MSCs in musculoskeletal injury. In local tissue damage, resident MSC-like cells in local tissue are first mobilized. However, the number of resident MSCs is limited. When more MSCs are needed for tissue repair, local chemokines will recruit more MSCs from the bone marrow, which is a complex multi-step process involving mobilization, homing, and repairation^36^, ^37^. Although we demonstrated that minimally invasive surgery can reduce inflammation, we need to further determine whether the decrease in mesenchymal cells infiltration rate is due to the reduced innate immune response or the absence of the adaptive immune response. Our study confirmed that the adaptive immune response (T cell immune response) was not present in the minimally invasive surgery group. Meanwhile, the innate immune response (granulocyte and monocyte immune response) was present in both groups to some extent. The aggregation characteristics of T cells in the material implantation area are correlated with mesenchymal cells infiltration and MSCs recruitment in terms of time and quantity. After 7 days of material implantation, T cells, mesenchymal cells, and MSCs began to appear in large quantities around the material, and other immune cells (innate immune cells) were not correlated with the recruitment of MSCs, which is consistent with existing studies^17^. B cells participate in the adaptive immune response by secreting immunoglobulin of various antigens; as such, material implantation is aseptic inflammatory and does not involve an antigen-antibody response. Therefore, in the study of biomaterial implantation, T cell immune response can represent adaptive immune responses to some extent^38^. The adaptive immune inactivation (T cell reduction) in the minimally invasive surgery group may be a key factor in the failure of osteoinduction of the biomaterial. In the context of fractures or bone defects, evidence indicates that T cell subsets inhibit bone regeneration.

For example, in Rag1-/- mice (mouse models without functional T cells or B cells), fracture healing accelerates^39^ with effector memory CD8 + T cells secrete IFN-γ and TNF-α to delay bone formation and fracture healing^40^. These results are contrary to the present findings. The discrepancy could be due to the high efficiency of the models of fracture or bone defect in bone remodeling environment with sufficient osteoblasts or MSCs, which are different from ectopia ossification in muscle tissues without sufficient niches of such bone stromal cells or stem cells. Therefore, other cells in the ectopia ossification microenvironment should release a large amount of chemokines to recruit MSCs for bone induction of biomaterials.

To confirm the role of T cells in tissue regeneration with biomaterials, we applied T cell depletion antibodies (secreted by hybrid tumor cells GK1.5 and 2.43) to the depletion of mouse CD3+ T cells. This method is one of the better recognized methods in the field, such as Rag1-/- genetically engineered mice (T cells and B cell dysplasia)^41^, ^42^. In addition, we also detected inhibited osteogenesis of biomaterials in nude mice compared with BALB/c **(Supplementary Fig. 6)**. The depletion of T cells is often used to study the function of T cells. Although both methods can achieve T cell loss or dysfunction, it has been reported that systemic T cell defects have significant effects on hematopoietic system and metabolism, which are important factors affecting bone repair in addition to the adaptive immune response^43^-^46^. As a result, we chose to use depletion antibodies to clear the T cells. Therefore, we used the injection of antibody every 3 days to ensure that the clearance efficiency of T cells in the model study stage was maintained at 90%. We were surprised to find that the depletion of T cells significantly reduced the infiltration of MSCs and other mesenchymal cells around the biomaterial and the osteogenic differentiation of MSCs, resulting in the loss of tissue regeneration effect of biomaterials, which is consistent with previous research^47^, ^48^. Other studies confirmed that T cells stimulated by inflammatory signals are recruited to the surrounding material and activate the NF-κB signaling pathway. These phenomena resulted in the enhanced release of chemokine CCL5 and recruitment of MSCs to the lesion^49^. In our study, we simulated the inflammatory environment of T cells *in vitro*, enhanced the NF-κB signaling pathway, and promoted the expression of CCL5 to achieve a strong chemotaxis on MSCs and bone induction effect of biomaterials. Based on the results of this study, despite that the minimal surgical trauma can guarantee a good prognosis and cause a smaller inflammatory response, it also reduces the recruitment effect of MSCs and the osteoinduction effect of biomaterials. Therefore, we suggest that the activation of inflammation should be selected during the implantation of clinical osteogenic materials, either by surgical means or by postoperative administration of drugs. In addition, MSCs and chemokines such as CCL5 can be used as potential factors for material modification in implant surgery where minimal inflammatory response must be pursued.

## Conclusion

Experimental and clinical studies have suggested that surgical trauma significantly affects the immune system. The degree of immune response caused by different surgical methods is not the same. With the development and application of implantable biomaterials, the cross dialogue between immune microenvironment and biomaterials cannot be ignored. At present, the development of biological materials or functional nanomaterials targeted at immune cells has gradually become a research hotspot in the field of tissue engineering. Such materials exhibit potential for treatment of some diseases. This study confirmed that the adaptive immune response, mediated by T cells, exerted recruitment effect on MSCs by using appropriate trauma surgical methods for material implantation. T cells are indispensable in material-induced tissue regeneration. Moreover, surgical trauma may determine the ultimate fate and therapeutic effect of biomaterials in experimental research and clinical application. These findings provide a new perspective for the rational use of surgical trauma to optimize tissue regeneration and target T cells to develop tissue engineering materials.

## Supplementary Material

Supplementary figures.

## Abbreviations

α-TCP: alpha-tricalcium phosphate
β-TCP: beta-tricalcium phosphate
ALP: alkaline phosphatase
ARS: Alizarin Red S
BCP: biphasic calcium phosphate
CaP: calcium phosphate
CCL5: chemokine C-C motif chemokine ligand 5
HA: hydroxyapatite
MSC: mesenchymal stem cell
NF-κB: nuclear factor-κB
PDTC: pyrrolidine dithiocarbamate
PMSF: phenylmethylsulfonyl fluoride
VEGF-α: vascular endothelial growth factor-α
