# Minimally invasive implantation and decreased inflammation reduce osteoinduction of biomaterial: Erratum

**DOI:** 10.7150/thno.70279

**Published:** 2022-01-21

**Authors:** Zifan Zhao, Qin Zhao, Bin Gu, Chengcheng Yin, Kailun Shen, Hua Tang, Haibin Xia, Xiaoxin Zhang, Yanbing Zhao, Xiangliang Yang, Yufeng Zhang

**Affiliations:** 1 State Key Laboratory Breeding Base of Basic Science of Stomatology (Hubei-MOST) and Key Laboratory of Oral Biomedicine, Ministry of Education, School and Hospital of Stomatology, Wuhan University, Wuhan 430079, China.; 2 Department of Dental Implantology, School and Hospital of Stomatology, Wuhan University, Wuhan, 430079, China.; 3 Institute of immunology, Shandong First Medical University & Shandong Academy of Medical Sciences, Taian 271000, China.; 4 National Engineering Research Center for Nanomedicine, Hubei Key Laboratory of Bioinorganic Chemistry and Materia Medica, College of Life Science and Technology, Huazhong University of Science and Technology, Wuhan 430074, China.; 5 Medical Research Institute, School of Medicine, Wuhan University, Wuhan 430071, China.

The authors apologize for the original versions of our paper unfortunately contained some incorrect representative images. We used the wrong representative images during figure assembly. The IHC images of 7dCon-Runx2, 10dCon-CD146/COL1A1 and 10dT-Del-Runx2 in Figure 6 have been misused. In addition, the horizontal coordinate of the bar chart Fig 6A is incorrectly marked, has been modified and corresponds to the images correctly. The correct versions of the Figure 6 were shown below.

The authors confirm that the corrections made in this erratum do not affect the original conclusions. The authors apologize for any consequences that these errors may have caused.

## Figures and Tables

**Figure 6:**
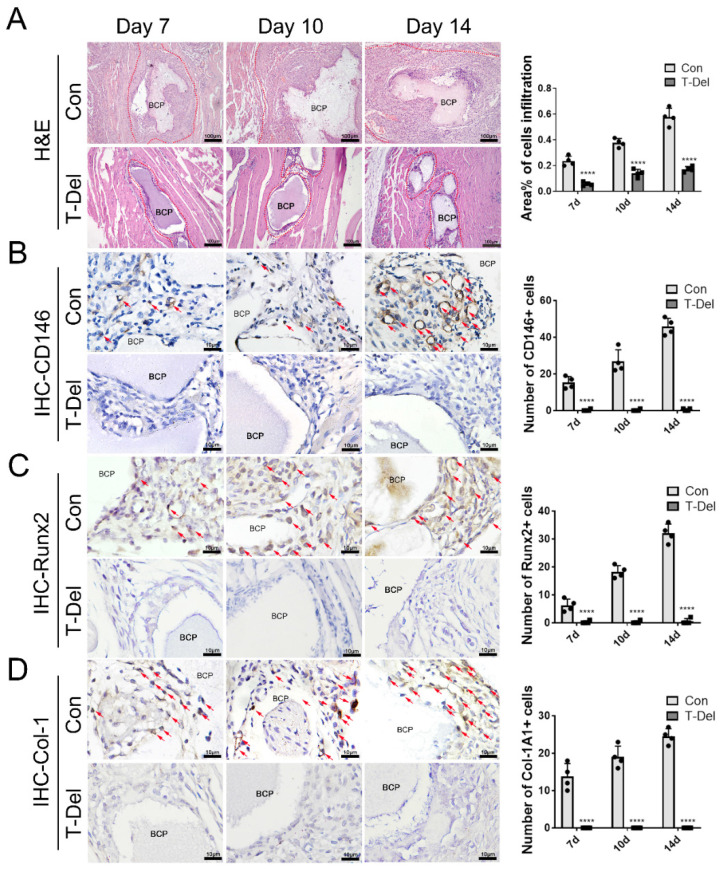
Infiltration of Cells and osteogenic differentiation MSCs around BCP dependent on T cells. (A) H&E staining showed cells infiltration was significantly reduced in the T-Del group. (B-D) IHC staining of CD146 (B), Runx2 (C), Col-1A1 (D) were detected and quantified by IHC staining. Positive expressions were significantly reduced in the T-Del group. (n=4) ****P < 0.0001.

