# Supplementary material for: Minimally invasive implantation and decreased inflammation reduce osteoinduction of biomaterial
Source: Theranostics. 2020 Feb 18;10(8):3533–45. doi: 10.7150/thno.39507 (PMC7069090; doi:10.7150/thno.39507)
Supplement: Supplementary file 1 — Supplementary figures. [file thnov10p3533s1.pdf]

Supplementary Figure 1

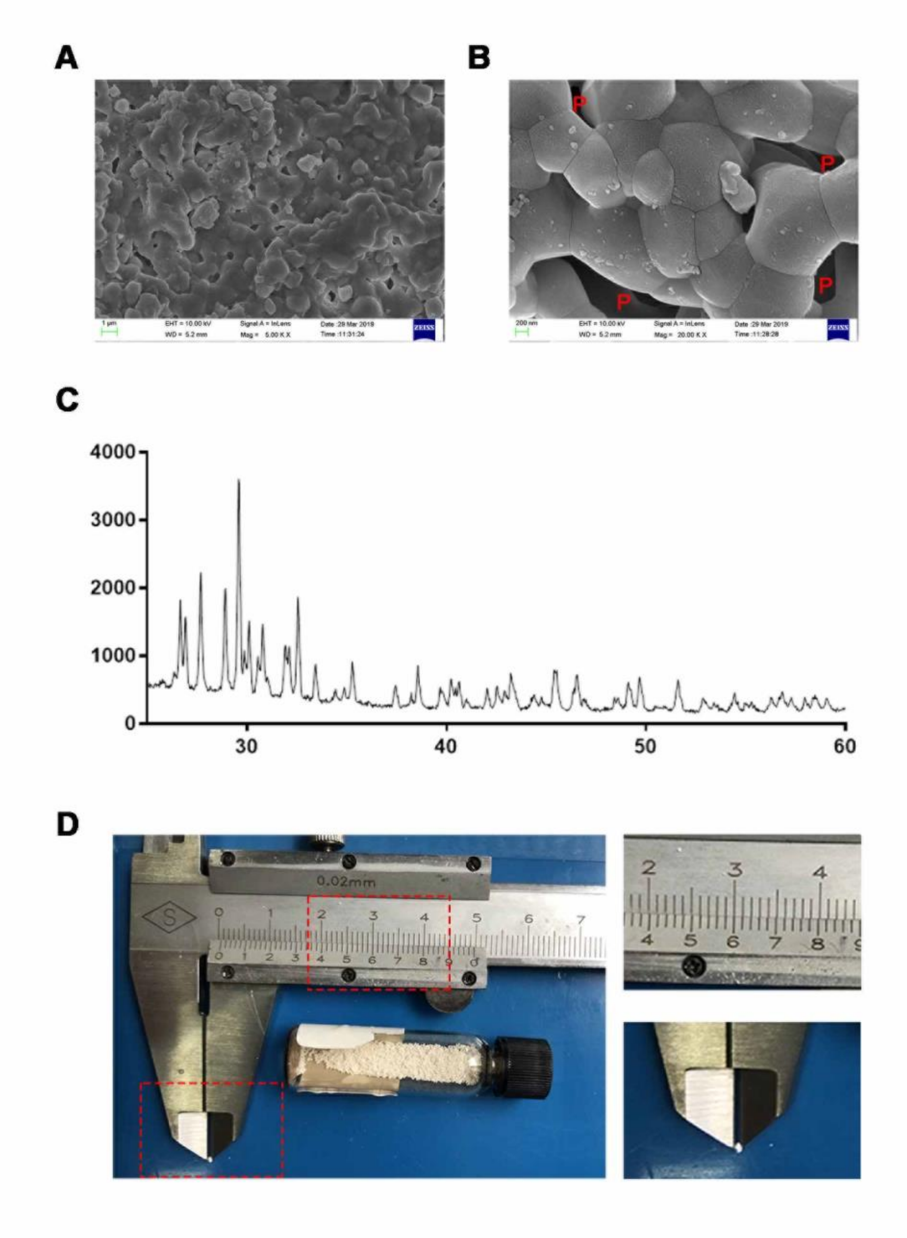

Scanning electron microscopy (SEM) micrographs (**A, B)** and X-ray diffraction (XRD) (**C)** for BCP granules. Macroscopic photo show dimensions of BCP granules (**D)**, BCP granules approximately 0.6mm in size were selected during implantation. P stands for pores.

Supplementary Figure 2

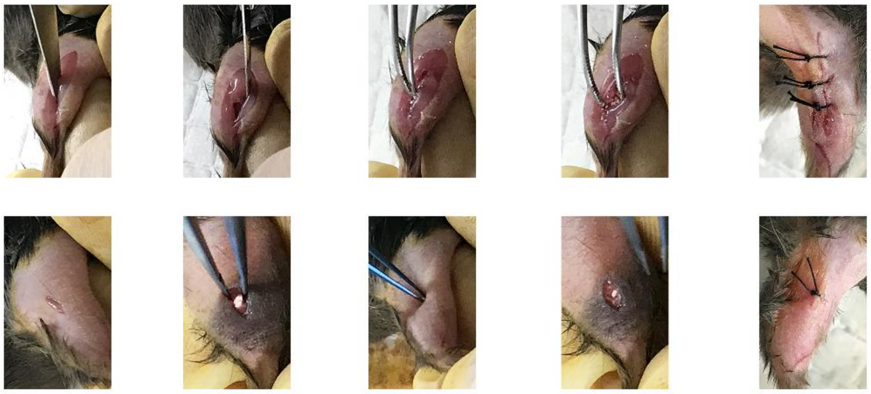

The processes of conventional incision and minimally invasive surgery.

Supplementary Figure 3

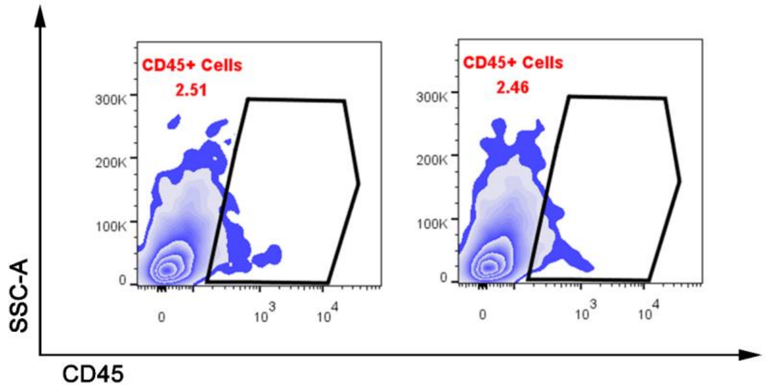

CD45+ immune cells in normal muscle tissue.

Supplementary Figure 4

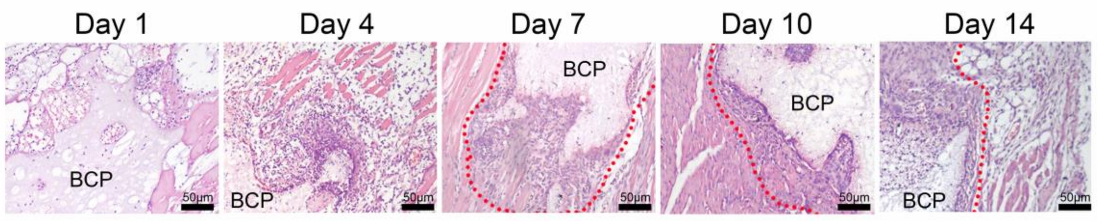

High power microscope images of infiltrating cells in Fig 3A.

Supplementary Figure 5

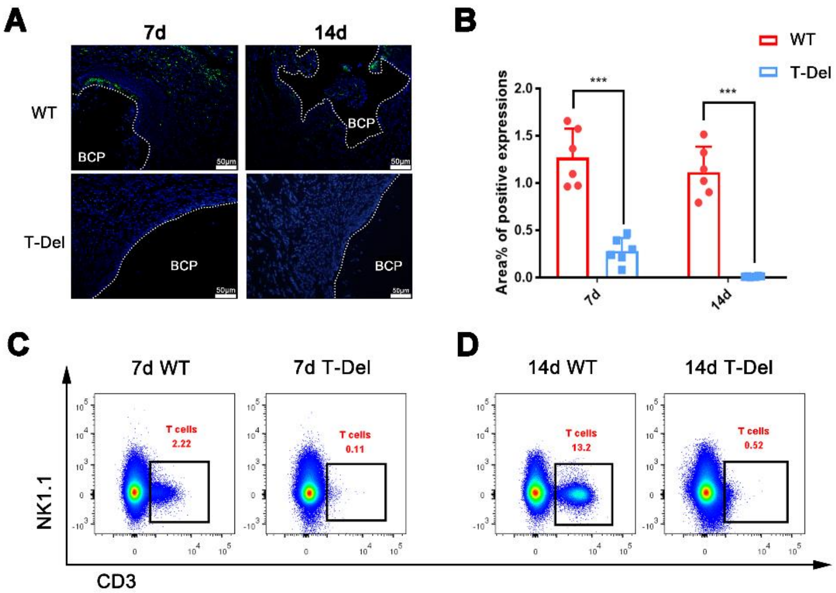

Deletion efficiency was determined by immunofluorescence (**A, B)** and flow cytometry (**C, D**) and the results were presented in (Supplementary Fig. 5), up to the experimental standard.

Supplementary Figure 6

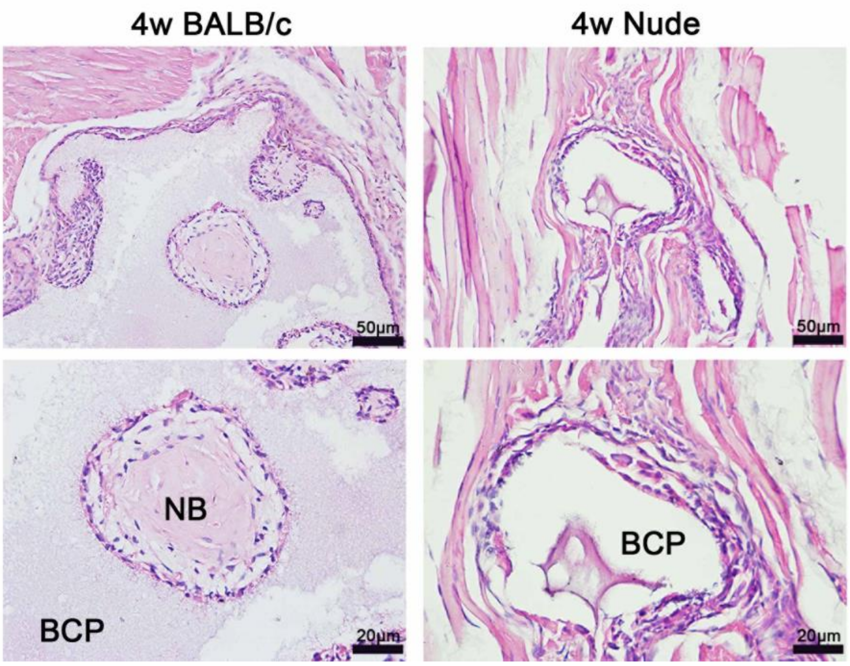

The osteoinduction material BCP implant in nude mouse also failed to heterotopic ossification.

Supplementary Figure 1

Scanning electron microscopy (SEM) micrographs (**A, B)** and X-ray diffraction (XRD) (**C)** for BCP granules. Macroscopic photo show dimensions of BCP granules (**D)**, BCP granules approximately 0.6mm in size were selected during implantation. P stands for pores.

Supplementary Figure 2

The processes of conventional incision and minimally invasive surgery.

Supplementary Figure 3

CD45+ immune cells in normal muscle tissue.

Supplementary Figure 4

High power microscope images of infiltrating cells in Fig 4A.

Supplementary Figure 5

Deletion efficiency was determined by immunofluorescence (Supplementary Fig. 5**A, B)** and flow cytometry (Supplementary Fig. 5**C, D**) and the results were presented in (Supplementary Fig. 5), up to the experimental standard.

Supplementary Figure 6

The osteoinduction material BCP implant in nude mouse also failed to heterotopic ossification.
